# Les perceptions des femmes tunisiennes selon le modèle des croyances liées à la santé et leurs pratiques relativement à l'ostéoporose

**DOI:** 10.11604/pamj.2016.23.42.6643

**Published:** 2016-02-15

**Authors:** Amina Belgacem, Amel Nouira, Sonia Soussi

**Affiliations:** 1 Centre Hospitalo-universitaire Sahloul, Sousse, Tunisie; 2 Département de Médecine Communautaire, Faculté de Médecine de Sousse, Tunisie; 3 Ecole Supérieure des Sciences et Techniques de la Santé de Tunis, Tunisie

**Keywords:** Osteoporosis, beliefs, behaviors, osteoporosis prevention, physical activity, calcium intake, Osteoporosis, beliefs, behaviors, osteoporosis prevention, physical activity, calcium intake

## Abstract

**Introduction:**

L'étude a pour objectif de décrire les croyances des femmes et leurs pratiques liées à la santé et à l'ostéoporose, afin d'élaborer des interventions efficaces et ciblées pour la prévention de cette maladie dans le contexte tunisien.

**Méthodes:**

Une étude descriptive transversale a été effectuée auprès de 100 femmes tunisiennes, âgées de 45 ans et plus, qui consultent au centre de santé de base d'une zone périurbaine de la région de Sousse (Tunisie). La collecte de l'information a été réalisée à l'aide de « l'échelle des croyances relatives à la santé sur l'ostéoporose» développée par Kim et ses collègues traduit en arabe et validé en Tunisie et le questionnaire de «Calcul des apports calciques quotidiens» développé par Fardellone Patrice. L'interprétation des résultants s'est basée sur le «Health Belief Model ».

**Résultats:**

La perception des participantes pourrait être considérée comme au dessus de la moyenne pour la vulnérabilité de l'ostéoporose (58%), la gravité de la maladie, les avantages de la pratique de l'activité physique, les avantages de l'apport en calcium et la motivation à la santé; par contre, elle pourrait être considérée comme modérée concernant les obstacles à la prévention. Cependant, les pratiques exposant au risque de la maladie sont relativement fréquentes et ceci essentiellement en rapport avec des facteurs socio-économiques et culturels.

**Conclusion:**

Les programmes de promotion doivent viser la création d'un environnement physique et social favorable à l'adoption des comportements à moindre risque et viser l'éducation ciblée de la population.

## Abstract (en)

**Introduction:**

The aim of this study is to describe beliefs and practices of women related to health and osteoporosis in order to develop effective and targeted interventions for the prevention of this disease in the Tunisian context.

**Methods:**

A descriptive cross-sectional study was conducted among 100 Tunisian women, aged 45 and older, who consult the basic health center in the suburban area of Sousse region (Tunisia). The collection of information was carried out with «osteoporosis health belief scale "developed by Kim and his colleagues validated and translated into Arabic in Tunisia and the questionnaire of "daily calcium inputs calculation" was developed by Patrice Fardellone. The interpretation of the results was based on the "Health Belief Model".

**Results:**

Perception participants considered above average for the vulnerability of osteoporosis (58%). The advantages severity of their benefits of calcium intake and health motivation could be considered moderate moderated. However, the practical exposure to disease risks is relatively frequent and essentially related to socio-economic and cultural factors.

**Conclusion:**

The promotion programs should aim at creating a supportive physical and social environment for the adoption of safer behaviors and especially education must be targeted.

## Introduction

A l'instar des pays à revenu intermédiaire, la Tunisie est concernée par une transition démographique et épidémiologique avec une augmentation des pathologies associées à l'amélioration de l'espérance de vie [1]. Selon l'Institut National de la Statistique (INS) en Tunisie, la proportion de la population âgée de 60 ans et plus, est de 10.1% et serait 20,1% en 2039 [2]. Cette situation démographique engendre des problèmes nouveaux et spécifiques liés à l'âge, dont l'ostéoporose, avec un besoin croissant de prise en charge sociale et sanitaire. En Tunisie, la prévalence de l'ostéoporose densitométrique post ménopause chez les femmes de plus de 50 ans, est de l'ordre de 23,4% [3]. En France, selon l'étude de Lespessailles et al, la prévalence globale de l'ostéoporose diagnostiquée est de 9,7% (8,6%-10,9%) et augmentait de façon linéaire avec l'âge [4]. 25% des femmes de 65 ans et 50% des femmes de 80 ans seraient atteintes d'ostéoporose [5]. Par ailleurs, l'ostéoporose est grave pour la santé par les complications et les handicaps qu'elle pourrait engendrer. La fréquence des fractures au niveau du col du fémur, des vertèbres, de l'humérus proximal et du poignet, survenant après un traumatisme mineur chez les femmes âgées de 50 ans et plus, dans un district urbain de la capitale Tunis est de 16,2% [6]. De même, en France, l'incidence des fractures augmente de façon exponentielle avec l'âge en rapport avec une diminution de la densité minérale osseuse qui constitue un facteur de risque de fracture [7]. Dans plusieurs régions du monde, les risques associés à l'ostéoporose chez les hommes sont environ la moitié de ceux des femmes [8]. En France, l'incidence de fractures ostéoporotiques est plus élevée chez les femmes que chez que les hommes, bien que la mortalité par fracture de la hanche soit plus importante chez les hommes [9]. Outre le sexe et l'âge avancé, d'autres facteurs de risque sont identifiés dont les antécédents familiaux d'ostéoporose, les antécédents personnels de fracture d'os fragile, la ménopause avant 45 ans, la consommation faible de calcium et de la vitamine D, la consommation excessive d'alcool ou de caféine, le tabagisme et la sédentarité [10, 11]. Néanmoins, les études ont souligné l'intérêt d'adopter des comportements favorables à la prévention de ce problème de santé. Ceci permettrait d'éviter ou de retarder la perte osseuses et ses complications, notamment les fractures [12]. Parmi les habitudes de vie les plus recommandées, l'exercice physique, le régime alimentaire ou les suppléments en calcium et en vitamine D en quantité adéquate, et l'absence du tabagisme ou le sevrage tabagique et l'absence ou la consommation modérée d'alcool et de la caféine [12–14]. Cependant, ces mesures préventives ne sont pas toujours adoptées par les femmes. En fait, selon le modèle des croyances liées à la santé ou Health Belief Model (HBM), l'approche psychosociale la plus utilisée depuis plus de trente ans pour expliquer les comportements de santé, les comportements de santé des individus sont en relation avec les perceptions de la menace à la santé et la perception des bénéfices de l'action à entreprendre [15]. Ainsi, on se demande quelles sont les perceptions et les pratiques des femmes tunisiennes dans une zone périurbaine relativement à l'ostéoporose? Ce travail permettra d'adapter les interventions de prévention au contexte périurbain de la Tunisie.

## Méthodes

Une étude descriptive transversale effectuée en 2013, a permis de recueillir les données par un enquêteur chercheur, auprès de 100 femmes tunisiennes qui consultent au centre de santé de base d'une zone périurbaine de 14380 habitants, de la région de Sousse (Tunisie). Deux instruments de collecte des données validés ont été utilisés après avoir eu l'autorisation des auteurs, soit "l'échelle des croyances relatives à la santé sur l'ostéoporose" (OHBS), développée par Kim et ses collègues [16] et le questionnaire de "Calcul des apports calciques quotidiens" développé par Fardellone Patrice [17]. La validation de contenu de ces outils et de leur version arabe a été obtenue à la suite d'un pré-test et la revue par un comité d'experts tunisiens dans ce domaine. Le traitement des résultats obtenus par l'OHBS est effectué par le calcul du score total et des scores partiels de chacune des 7 sous échelles de l'instrument à savoir la vulnérabilité à l'ostéoporose, la gravité de la maladie, les avantages de la pratique de l'activité physique, les avantages de l'apport en calcium, les obstacles à la pratique de l'activité physique, les obstacles à la consommation de calcium et enfin la motivation à la santé. Le score total pourrait varier entre 42 et 210 et les scores partiels de chaque sous échelle pourrait varier de 6 à 30. Pour la majorité, les scores des sous-échelles les plus élevés indiquent des croyances très positives. Mais pour les deux sous-échelles sur les obstacles, les scores les plus élevés indiquent les croyances de santé les plus négatifs. Le calcul des apports calciques quotidiens est réalisé à l'aide du calculateur du « Groupe de recherche et d'information sur l'ostéoporose» (Grio) disponible en ligne au www.grio.org. Une ration calcique est jugée satisfaisante si elle est supérieure à 1200mg/jour. La discussion des résultants s'est basée sur le «Health Belief Model ». Les considérations éthiques ont été respectées à travers l'approbation de l'étude par le comité local d'éthique de la recherche, le respect de la confidentialité et l'obtention du consentement libre et éclairé des enquêtées.

## Résultats

Au total, cent femmes ont été incluses à l'étude. Elles sont toutes de race blanche et âgées de 45 ans et plus, avec une moyenne d'âge de 57,79 ± 9,73 ans. Elles ont plutôt un niveau socioéconomique moyen et un niveau de scolarité bas (89% analphabètes ou niveau primaire); la majorité des participantes sont des femmes au foyer (82%) et elles sont ménopausées (86%). La ménopause précoce est notifiée chez 38% des femmes ménopausées. 22% des femmes présentent un Indice de Masse Corporel (IMC) normal, les autres femmes sont soit obèses (36%), soit présentant un surpoids (35%). Des antécédents familiaux d'ostéoporose sont notés chez 21% des enquêtées. Concernant les perceptions du risque, plus de la moitié des femmes (58%) perçoivent qu'elles sont susceptibles à l'ostéoporose et les trois quarts considèrent que la maladie est grave. En effet, elles jugent que le fait d'avoir la maladie affecterait leur perception de soi (82%), l'effraie (77%), lui couterait très cher (76%), l'handicaperait et serait une source de dépression (70%). Concernant les bénéfices de la prévention, la plupart des femmes perçoivent que l'activité physique serait bénéfique (93%), les satisferait (92%), aiderait à renforcer les os (88%), protègerait contre les problèmes causés par l'ostéoporose (87%), les permettrait de se sentir mieux (80%) et atténuerait le risque d'avoir des os fragiles (75%). De même, les femmes perçoivent que la consommation de calcium les satisferait (95%), serait très bénéfique (80%) et réduirait l'inquiétude de la maladie (78%). Cependant, les femmes perçoivent des obstacles à la prévention. Les trois quarts signalent que la pratique de l'activité physique est difficile en l'absence de lieu approprié pour pratiquer du sport (78 %), qu'elles ne sont pas encouragées de la part de leur mari ou famille (42%) et que la pratique régulière du sport demande un nouveau style de vie, ce qui n'est pas facile (38%) et ce qui pourrait perturber leur mode de vie (21%). Parmi les obstacles de consommation de calcium, le coût des aliments riches en calcium est jugé élevé par la plupart de femmes (88%). Par ailleurs, l'augmentation de consommation de ces types d'aliments les appellent à sacrifier d'autres aliments qu'elles aiment (76%). Néanmoins, autour des trois quarts des femmes déclarent qu'elles supportent les aliments riches en calcium (72%) et qu'elles les préfèrent (70%). La majorité des femmes montre une motivation positive pour la santé. En effet, 89% d'entre elles affirment qu'elles sont à l'écoute de nouvelles informations concernant la santé et priorisent la bonne santé. Egalement, 81 % des participantes ont tendance à découvrir prématurément leurs problèmes de santé et suivent bien les recommandations pour se maintenir en bonne santé. Selon nos résultats, le score total d'OHBS est en moyenne de 143.75 ± 6.34 avec des extrêmes de 128 à 156 (Tableau 1). Les sous- scores sont de 19.29 ± 4.39 pour la perception de la vulnérabilité, de 21.38 ± 3.83 pour la perception de la gravité de la maladie, de 23.01 ± 1.83 pour la perception des avantages de la pratique de l'activité physique, de 22.73 ± 2.13 pour la perception des avantages de l'apport en calcium, de 17.18 ± 3.22 pour la perception des obstacles à la pratique de l'activité physique, de 18.93 ± 2.84 pour la perception des obstacles à l'apport en calcium et de 21.27 ± 2.21 pour la perception de la motivation à la santé (Tableau 1).

**Tableau 1:** Les Sous échelles des croyances relatives à la santé sur l'ostéoporose (n = 100)

Sous échelle OHBS	Score possible	Minimum	Maximum	Moyenne	Ecart-type
Susceptibilité / Vulnérabilité	6-30	12	24	19.29	4.39
Gravité / Sévérité	6-30	11	26	21.38	3.83
Avantages de l'activité physique	6-30	17	25	23.01	1.83
Avantages de l'apport en calcium	6-30	18	28	22.73	2.13
Obstacles de l'activité physique	6-30	11	24	17.18	3.22
Obstacles de consommation de calcium	6-30	12	24	18.93	2.84
Motivation à la santé	6-30	18	30	21.27	2.21

Score total d'OHBS = 143.75 ± 6.34

Ainsi, la perception des participantes pourrait être considérée comme au dessus de la moyenne pour la vulnérabilité de l'ostéoporose, la gravité de la maladie, les avantages de la pratique de l'activité physique, les avantages de l'apport en calcium et la motivation à la santé; par contre, elle pourrait être considérée comme modéré concernant les obstacles à la pratique de l'activité physique et les obstacles à la consommation de calcium. En effet, les participantes qui évaluent leur risque aussi bas, elles les attribuent principalement à leurs propres comportements de prévention, essentiellement la consommation de calcium et la pratique de l'activité physique, tandis que les femmes qui évaluent leur risque plus élevé, elles l'attribuent principalement à leur histoire familiale. Par ailleurs, les pratiques influant la survenue de l'ostéoporose sont relativement fréquents, à savoir l'absence d'une pratique d'une activité physique régulière (21% des femmes pratiquent la marche), l'apport calcique insuffisant (100%), la faible exposition solaire quotidienne (en moyenne 57,97 minutes ± 551,8 par jour), un port fréquent du voile (91%). Par ailleurs, la consommation de caféine est de 23%, le tabagisme est très faible (2%) et l'alcoolisme est absent. La consommation quotidienne du lait et la consommation hebdomadaire des yaourts et des fromages ne sont notées que chez une femme sur cinq. La consommation quotidienne de la viande et du poisson est signalée par un tiers des femmes, avec des portions plutôt moyennes à raison d'une fois par jour. Par contre, la consommation des pâtes ou de la semoule est quotidienne, plutôt en grandes quantités et celle des œufs et des pommes de terre est en moyenne de l'ordre de 3 à 4 fois par semaine. Par ailleurs les enquêtés consomment très peu de fruits, plutôt l'eau de robinet (80%) et pas du tout de chocolat. Il est à noter que les femmes sont capables d'identifier les principales sources alimentaires de calcium. Concernant la ration calcique, les apports quotidiens varient de 214 à 774 mg par jour avec une moyenne de 470,16 ± 143,22 mg/j, soit des apports inférieurs aux besoins recommandés (>1200mg/j). La proportion des femmes ayant une ration calcique quotidienne inférieure à 600 mg est respectivement de 84% et de 76%, chez les âgées de 45 à 50 ans et celles âgées de plus de 50 ans.

## Discussion

D'une façon globale, les perceptions des femmes enquêtées semblent plutôt modérées aussi bien relativement aux perceptions du risque de l'ostéoporose que celle aux bénéfices de la prévention. Par ailleurs, généralement les pratiques ne sont pas en faveur de la prévention de l'ostéoporose. Nous jugeons que les résultats de l'étude sont valides puisque les biais d'information ont été prévenus grâce à l'utilisation des instruments de mesure validés et par le fait que la collecte de l'information a été réalisée par un seul chercheur. L'usage de deux instruments de mesure nous a permis de recouper interpréter les données. Néanmoins, les résultats ne peuvent être généralisés, mais elles donnent un aperçu sur une frange large de la population sachant qu'en Tunisie, les zones périurbaines sont de plus en plus étendues avec le mouvement d'exode rural vers les grandes villes. En fait, le Health Belief Model (HBM) qui est centré sur les perceptions pour expliquer les comportements, postule qu'un individu adopte un comportement s'il est conscient de la gravité du problème, s'il se sent concerné, si le comportement à adopter présente pour lui plus des avantages que des inconvénients et s'il se croit capable de le réaliser [15]. Relativement à la perception de la susceptibilité, près de la moitié des femmes enquêtées se perçoivent non susceptibles à l'ostéoporose. Ces résultats corroborent avec ceux de. Gerend MA et al. qui notifient que les femmes âgées de 40-86 ans croient qu'elles sont moins susceptibles de développer l'ostéoporose que les autres femmes de leur âge [18]. La perception du risque de l'ostéoporose est moins importante que celle du cancer du sein et des maladies cardiaques [19–22]. Néanmoins, les étudiants américains perçoivent qu'ils ont une forte chance d'être atteints par l'ostéoporose et cette prise de conscience a modifié leurs comportements [20]. Ces divergences seraient en rapport avec les actions de prévention menées dans les différents pays. En fait, dans notre population, les niveaux socio économique et éducationnel des enquêtées sont plutôt faibles. En fait, ces facteurs sont jugés les facteurs les plus prédicateurs de l'ostéoporose [21]. La perception de la sévérité qui reflète la perception de la menace et des conséquences néfastes de la maladie, est une des composantes de HBM qui influence la manière de considérer l'ostéoporose. Les trois quarts des femmes de notre étude (74%) croient que l'ostéoporose aurait une influence significative sur leur vie. D'autres études ont confirmé que plus de 80% des enquêtées croit que l'ostéoporose est une maladie grave [22, 23]. De même, les étudiants américains ont une croyance élevée que l'atteinte par l'ostéoporose est grave; l'idée d'être atteinte par cette maladie les effarerait et affecterait leurs perceptions [20]. A la Nouvelle-Zélande, les femmes ont une faible perception de leur susceptibilité de développer l'ostéoporose, mais la majorité la considère comme une maladie sérieuse [24]. Selon le HBM, à côté de la perception de menace (susceptibilité et sévérité), le comportement serait le résultat aussi de la perception des avantages et des obstacles aux actions préventives à entreprendre. L'activité physique et la consommation suffisante du calcium sont parmi les mesures les plus recommandées de prévention de l'ostéoporose [12–14]. Bien que les enquêtées de notre étude aient une vision positive des avantages de la pratique de l'activité physique et qu'elles se sentent satisfaites d'elles-même en pratiquant du sport en prévention de l'ostéoporose, l'activité physique n'est pratiquée d'une façon régulière que par 21% des femmes. Le degré de motivation et les aptitudes des individus sont des facteurs individuels qui influencent l'adoption d'activité physique [25]. En outre, l'environnement physique, social et culturel influence la volonté de la femme à entreprendre une activité physique régulière. En effet, 78% des femmes de notre étude ont justifié leur inactivité physique par le manque de lieu approprié pour pratiquer du sport. Dans le même sens, Yin-Ping Zhang et al. ont suggéré que le manque d'espace est un obstacle à l'activité physique [26]. Par ailleurs, le contexte tunisien et notamment dans les zones à moyen et à bas niveaux socio-économiques, les femmes ne sont pas encouragées autant que les hommes à pratiquer une activité sportive, c'est le cas de 42% des enquêtées. En effet, Pender a montré que l'activité physique n'est pas considérée comme une activité appropriée pour la femme dans certains groupes ethniques [27]. Par ailleurs, une fois adopté, un comportement devrait être maintenu. En fait, la perception de l'auto-efficacité constitue un facteur prédictif fiable de maintien du comportement relatif à la pratique de l'activité physique [28]. L'apport calcique suffisant constitue un autre facteur de prévention de l'ostéoporose. En fait, plusieurs études soutiennent que la consommation de calcium alimentaire et de vitamine D améliore la masse osseuse chez les personnes ayant une faible densité osseuse et qu'elle réduit les risques d'ostéoporose , des chutes et des fractures périphériques [29]. Les femmes enquêtées y croyaient et elles sont capables d'identifier les sources de calcium. Nos résultats corroborent avec ceux de l'étude effectuée auprès des étudiants américains qui ont des fortes croyances à propos de la relation entre l'apport en calcium et la réduction du risque d'ostéoporose et des fractures osseuses [20]. Selon le rapport de l'Institute of Medicine (IOM) des États-Unis, l'apport de calcium recommandé pour les femmes adultes 45-50 ans est de 1000 mg/j et pour les femmes âgées de plus de 51 ans est de 1200 mg/j [30]. Cependant, dans notre étude, l'apport calcique est insuffisant pour toutes les femmes avec une ration calcique moyenne de 470,16 ± 143,22 mg/j (214 - 727). D'autres études ont souligné l'insuffisance des apports calciques auprès des femmes mais à des proportions moins importantes à savoir, 58% des filles canadiennes âgées de 12 à 16 ans [31] et 89% des femmes iraniennes âgées de plus de 45 ans [32]. En France, 37,2 % des femmes ménopausées âgées de plus de 45 ans consomment moins de 600 mg/jour et seulement 20,1 % ont une consommation journalière supérieure à 1000 mg/jour. En outre, la proportion de femmes consommant moins de 600 mg/jour augmente avec l'âge [33]. Dans notre étude, la perception des obstacles à l'apport de calcium est moyenne. En fait, le coût de nourriture riche en calcium a été jugé élevé vu leur niveau socio-économique. Ceci explique en partie la faible ration calcique consommée par les femmes. Les étudiants chinois jugent aussi que les aliments riches en calcium sont chères et que l'augmentation de consommation d'aliments riches en calcium les appelaient à sacrifier d'autres aliments qu'ils aimaient [20]. La perception de la motivation à la santé influence aussi les comportements. Les femmes de notre étude ont des idées et des comportements en accord avec les items relatifs à la motivation positive pour la santé. Cela a été aussi constaté par d'autres études [20, 22]. La prévention de l'ostéoporose qui est le moyen le plus rentable pour gérer l'ostéoporose [34], appréhende essentiellement des stratégies pour maximiser le pic de masse osseuse dès l'adolescence et pour minimiser les pertes osseuses en luttant contre les facteurs de risque modifiables [35, 36]. Le programme de prévention de l'ostéoporose utilisant le modèle de croyance liée à la santé conçu par Turner. L.W et al. inspiré de la Fondation Internationale de l'Ostéoporose est basé essentiellement sur des programmes de promotion et d'éducation de la santé [36].

## Conclusion

Dans le contexte tunisien, les programmes d'éducation pour la santé sont envisageables d'autant plus que l'on dispose d'une bonne couverture du territoire par les structures de 1ère ligne sachant que plus de 90% de la population habitent à moins de 5 km d'un CSB et que la médecine scolaire est assez développée avec une couverture totale des établissements scolaires et universitaires par les équipes médicales. Néanmoins, pour réduire effectivement le risque et la fréquence de l'ostéoporose, il faut créer un environnement approprié à l'adoption des comportements favorables à la santé et à la prévention de l'ostéoporose, avec un engagement politique et une collaboration avec les différents secteurs y compris celui de l'industrie alimentaire et tout en améliorant le niveau socio-économique. La recherche permettra de cibler et d'adapter les interventions et d'évaluer les actions de prévention de l'ostéoporose.

### Etat des connaissances sur le sujet

- Selon le modèle des croyances liées à la santé, les comportements de santé des individus pour la prévention de l'ostéoporose sont en relation avec les perceptions de la menace à la santé et la perception des bénéfices de l'action à entreprendre.

### Contribution de notre étude a la connaissance

- Les perceptions des femmes enquêtées semblent plutôt modérées aussi bien relativement aux perceptions du risque de l'ostéoporose que celle aux bénéfices de la prévention.
- Les pratiques ne sont pas en faveur de la prévention de l'ostéoporose et ceci essentiellement en rapport avec des facteurs socio-économiques et culturels.
- Pour réduire effectivement la prévalence de l'ostéoporose, il faut créer un environnement approprié à l'adoption des comportements favorables à la santé des os et il est important d'encourager l'éducation de la santé.
